# Primary signet-ring cell carcinoma of the prostate involving the pelvis: a case report

**DOI:** 10.3389/fonc.2024.1444541

**Published:** 2024-12-11

**Authors:** Shuai Luo, Xiaoxue Tian, Ting Xu, Jinjing Wang

**Affiliations:** Department of Pathology, Affiliated Hospital of Zunyi Medical University, Zunyi, Guizhou, China

**Keywords:** signet-ring cell carcinoma, prostate cancer, pelvic puncture, cytology, histopathologic

## Abstract

**Background:**

Signet-ring cell carcinoma (SRCC) originates from undifferentiated stem cells in the neck of glands within the lamina propria of the mucosa. Primarily affecting the stomach, SRCC can also involve the breast, pancreas, gallbladder, colon, and bladder, although these cases are rare. SRCC of the prostate is extremely rare, and diagnosing it *via* pelvic puncture is particularly challenging. Prostate SRCC is a distinct type of malignant tumor characterized by unique biological behavior, high malignancy, rapid disease progression, and poor prognosis. Due to its rarity, early diagnosis and treatment are critical. Currently, the diagnosis and treatment of this disease present significant challenges.

**Case demonstration:**

A 74-year-old male patient was admitted to our hospital with “left lower abdominal pain, changes in bowel habits, and bloody stools for 2 months.” A contrast-enhanced pelvic CT scan revealed a soft tissue density mass on the left side of the pelvis. Contrast-enhanced Pelvic MRI suggested a tumor with a rich blood supply on the left side of the pelvis, indistinguishable from the left seminal vesicle and prostate gland, indicating the presence of a suspected malignant tumor. Pathologic biopsy of the pelvic mass confirmed the diagnosis of prostate SRCC. The patient subsequently underwent chemoradiotherapy and has been followed up for three months. He is currently in good condition.

**Conclusion:**

SRCC predominantly occurs in the digestive tract and rarely originates in the prostate. Diagnosing prostate SRCC through abdominal paracentesis is challenging. To the best of our knowledge, this is the first reported case of SRCC of the prostate initially presenting with gastrointestinal symptoms. Additionally, it presents a case of prostate SRCC involving the pelvis, confirmed through pelvic puncture. highlighting its significance for clinical diagnosis.

## Background

Signet-ring cell carcinoma (SRCC) predominantly occurs in the gastrointestinal tract and has a low incidence in the pancreas, breast, thyroid, and bladder. SRCC originating in the prostate is extremely rare (1). Primary prostate SRCC is prevalent in elderly male patients, presenting with clinical symptoms such as bladder outlet obstruction, dysuria, dyspareunia, nocturia, and urinary retention. Some patients may also experience bladder irritation and hematuria (2). Serum PSA levels may be elevated or within the normal range. SRCC is a unique type of mucus-secreting adenocarcinoma characterized by abundant cytoplasm, a nucleus squeezed to one side, and a “signet-ring” appearance due to the presence of full mucus in the cytoplasm. The diagnosis of primary prostate SRCC relies primarily on histopathologic examination. Fujita et al. (3) reported 3- and 5-year survival rates of 50% and 25%, respectively, for patients with prostate SRCC. Therefore, early diagnosis and timely treatment with radical prostatectomy, endocrine therapy, and chemoradiotherapy are crucial for improving patient survival rates.

Unlike previous reports, this is the first documented case of SRCC initially manifesting with gastrointestinal symptoms, offering valuable insights for clinicians to enhance diagnostic approaches and reduce the risk of misdiagnosis.

## Case demonstration

A 74-year-old male patient was admitted to our hospital with “left lower abdominal pain, changes in bowel habits, and bloody stools for 2 months.” and had no previous history of major surgery or cancer. Two months earlier, he experienced left upper abdominal pain accompanied by changes in bowel habits, with bowel movements occurring once a week and producing soft, formed stools. He reported no symptoms such as dizziness, palpitations, fatigue, urinary frequency, urgency, or hematuria. An abdominal CT at a local hospital revealed soft tissue masses in the pelvis, and an abdominal ultrasound suggested a hypoechoic mass. The patient was admitted to our hospital with a diagnosis of “pelvic tumor,” having lost 5 kg since disease onset.

Special examination showed slight abdominal distension, no gastrointestinal type and motor waves, no palpation of liver and spleen, no tenderness or rebound pain in the whole abdomen, slight tension in the whole abdomen, slight tenderness in the left lower abdomen, no hernia in the bilateral inguinal region, normal bowel sounds, and no percussion pain in the bilateral renal region. There was no malformation of the external perineal genitalia. A digital rectal examination revealed an enlarged prostate with palpation of a firm nodule.

**Figure 1:**
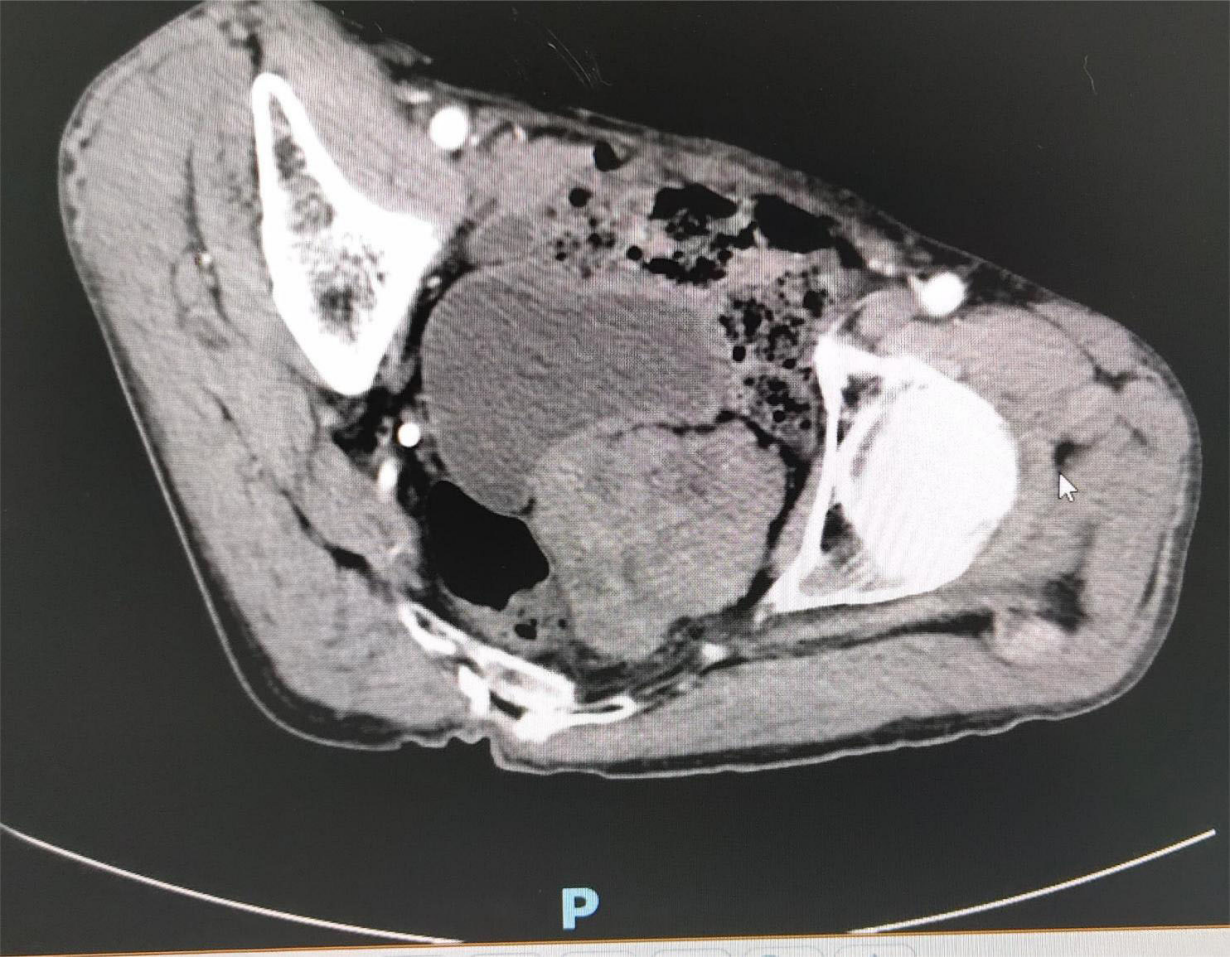
Pelvic CT showing a soft tissue density mass in the left portion of the pelvis, poorly demarcated from the left seminal vesicle glands and prostate, with an unclear border and uneven density.

**Figure 2:**
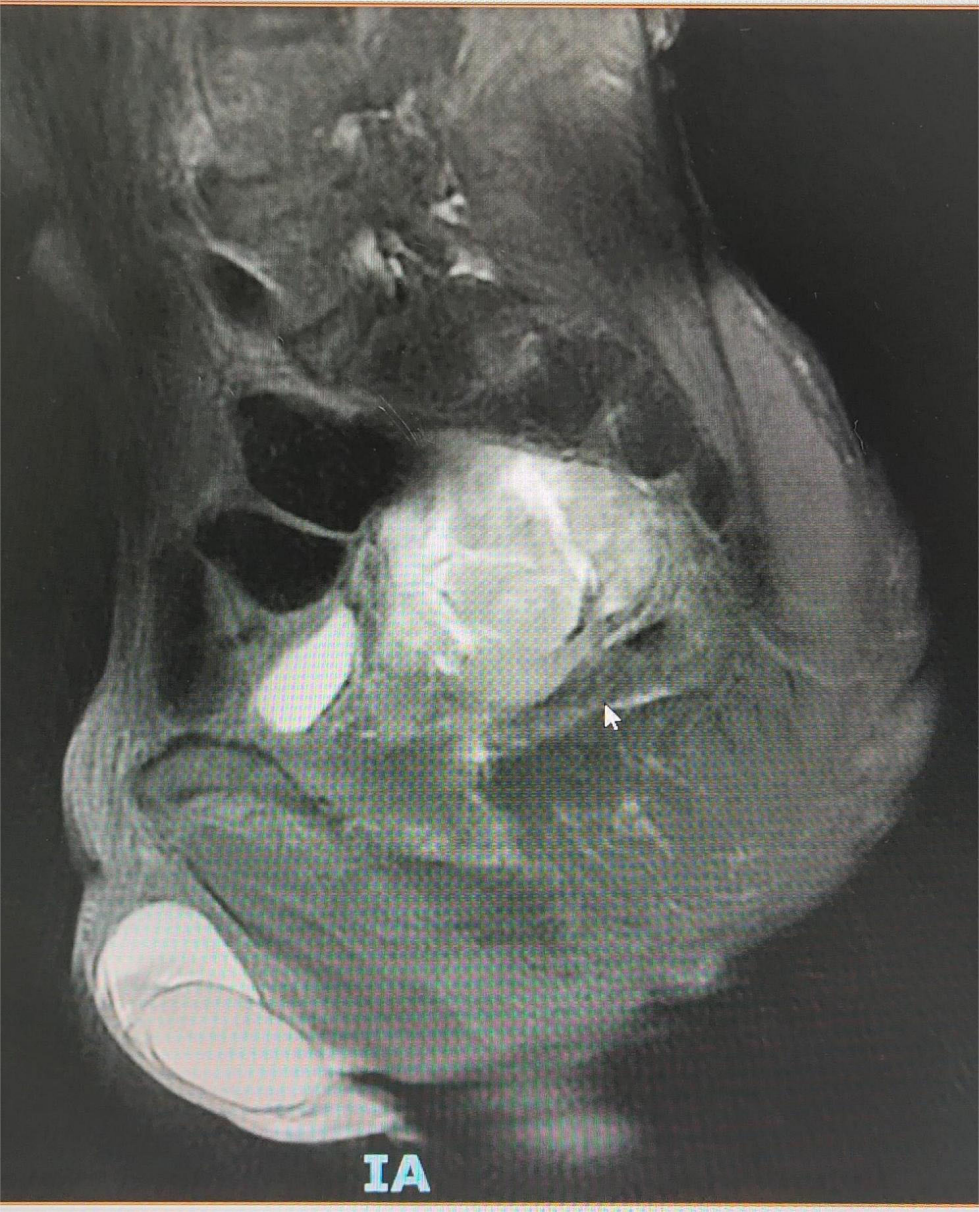
Pelvic MRI showing a blood-rich tumor-like lesion on the left side of the pelvis, poorly demarcated from the left seminal vesicle gland and prostate.

Contrast-enhanced pelvic CT scan (**Figure 1**) revealed a soft tissue density mass in the left pelvis, poorly demarcated from the left seminal vesicle gland and prostate, with a maximum cross-section of approximately 73 × 61 mm. The mass had an unclear border, uneven density, multiple patchy hypodensities, and an obviously uneven enhancement pattern on the scanned image. A contrast-enhanced pelvic MRI (**Figure 2**) revealed neoplastic lesions in the left pelvic area, characterized by a rich blood supply and poor demarcation from the left seminal vesicle glands and prostate, suggesting malignancy. Chest CT and bone imaging ECT showed no distant metastasis.

**Figure 3:**
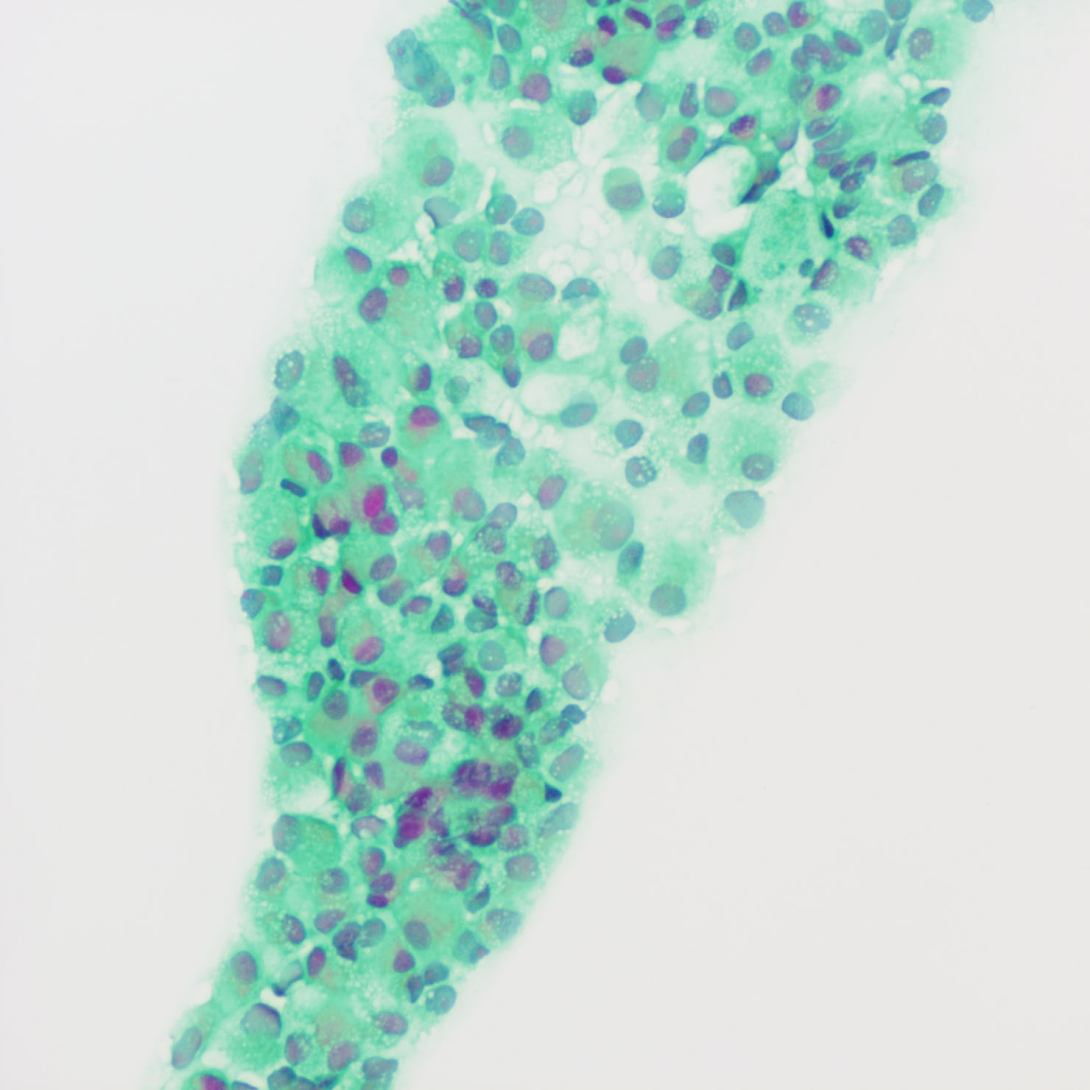
Smear test showing diffuse patches of tumor cells.

**Figure 4:**
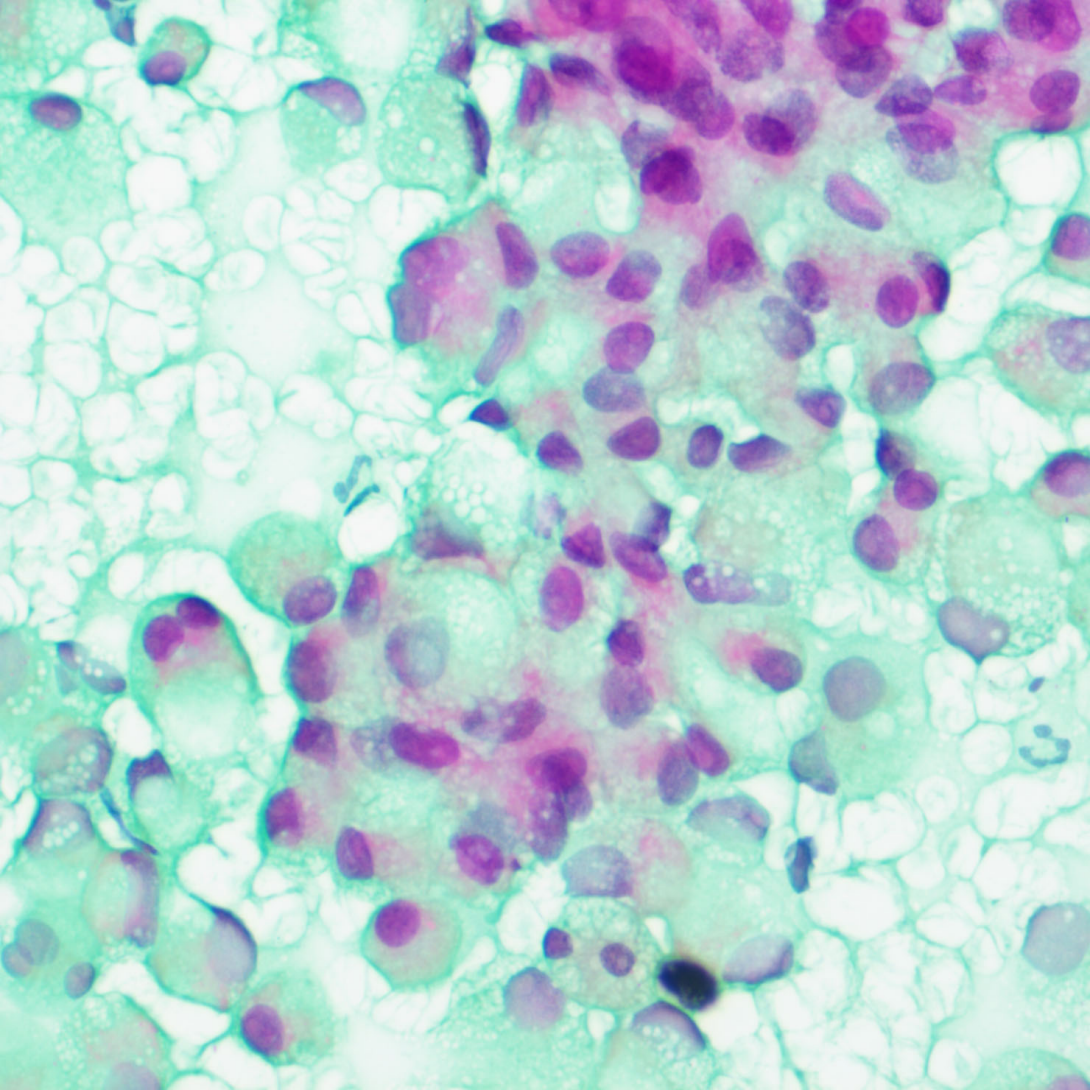
Smear test showing nuclei squeezed to one side by intracellular mucus, cells with a signet-ring appearance, denser chromatin, large deeply stained nuclei, and irregular nuclear membranes.

A tumor puncture cytobrush smear test performed after admission revealed diffuse patches of tumor cells in the smear (**Figure 3**). The nuclei were displaced to one side by intracellular mucus, imparting the cells a ring-like appearance with dense chromatin, large deeply stained nuclei, and irregular nuclear membranes (**Figure 4**). Part of the cytoplasm appeared cloudy, with occasional large mucus-secreting vacuoles, and the cytoplasmic borders were well-defined. Based on the cytological smear test, the diagnosis was as follows: (pelvic tumor puncture smear) signet-ring heterogeneous cells were detected, suggesting a neoplastic lesion. A biopsy was recommended for further clarification.

**Figure 5:**
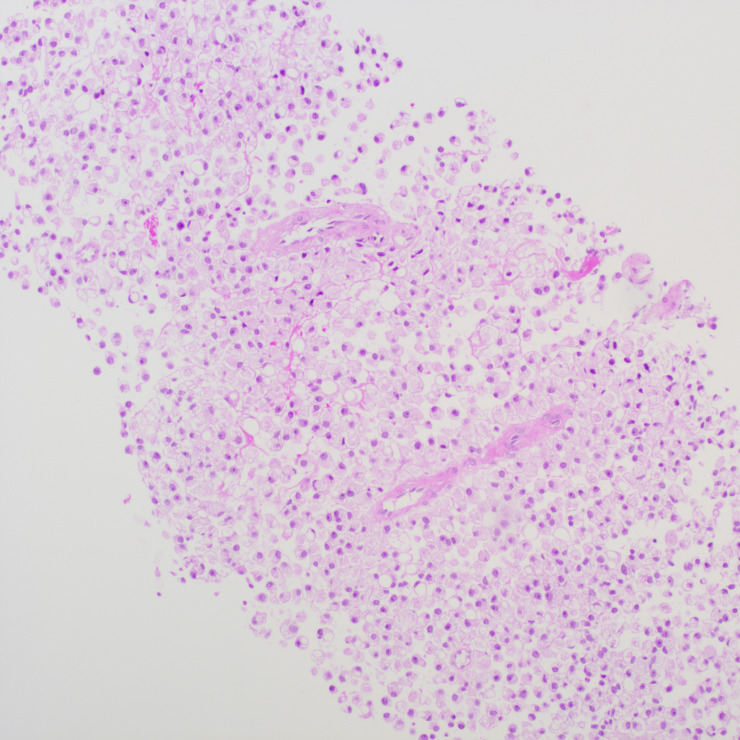
Pelvic puncture biopsy at low magnification showing diffuse patches of signet-ring cells in the punctured tissue.

**Figure 6:**
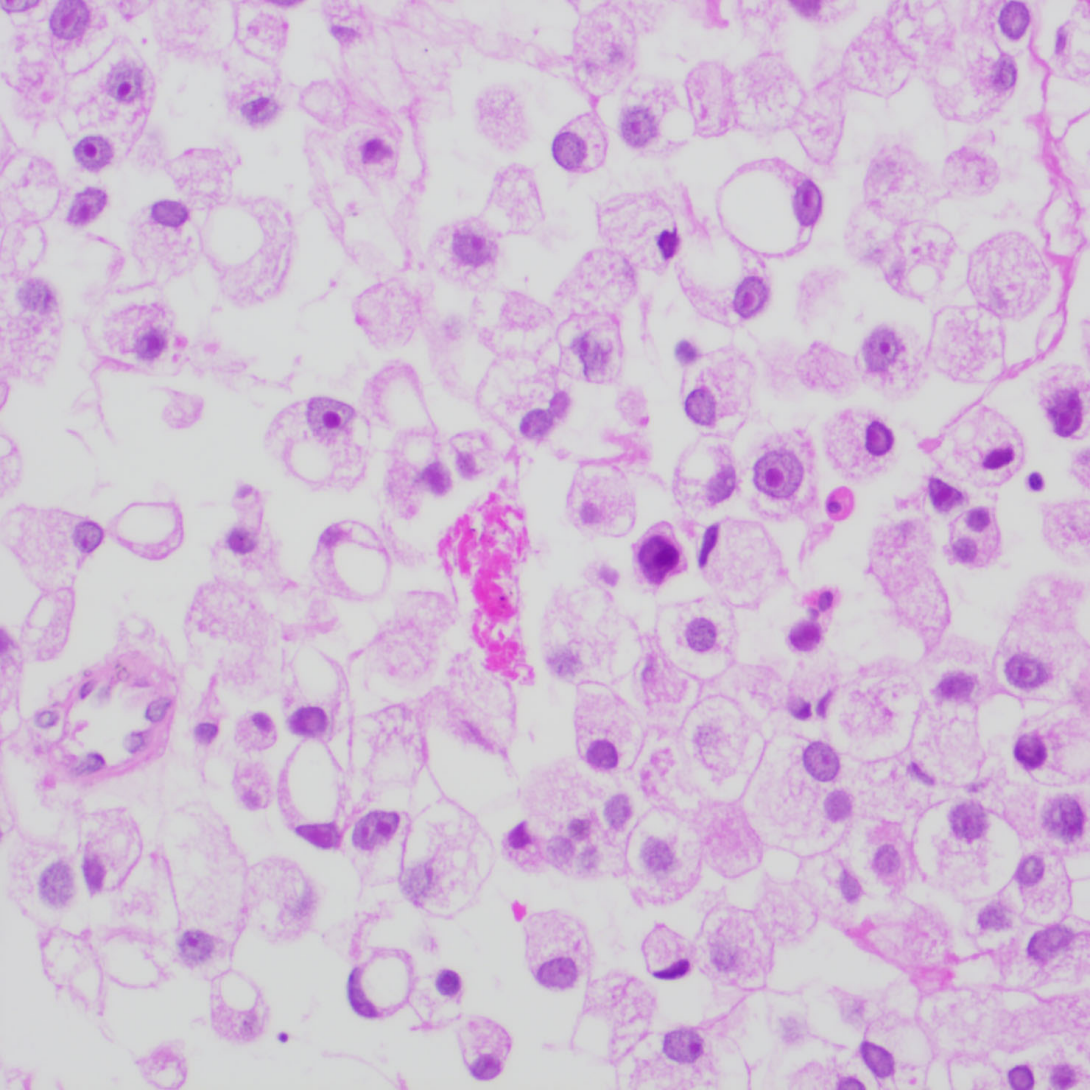
High magnification of tumor cells with nuclei crowded to one side, exhibiting a signet-ring appearance. Tumor cell nuclei are large and deeply stained, with dense chromatin, irregular nuclear membranes, and clear cytoplasmic boundaries.

**Figure 7:**
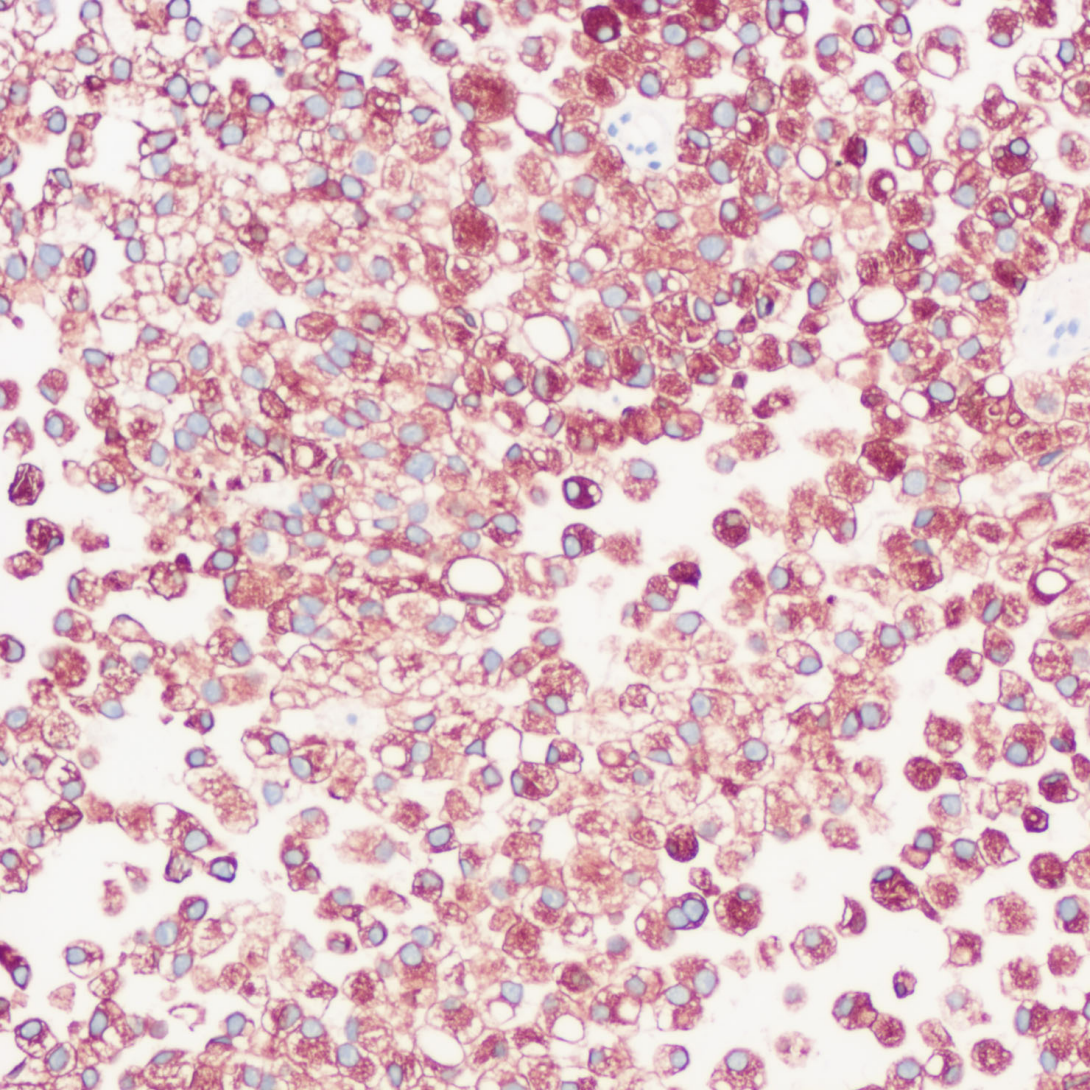
Immunohistochemistry showing CK positivity in tumor cells.

**Figure 8:**
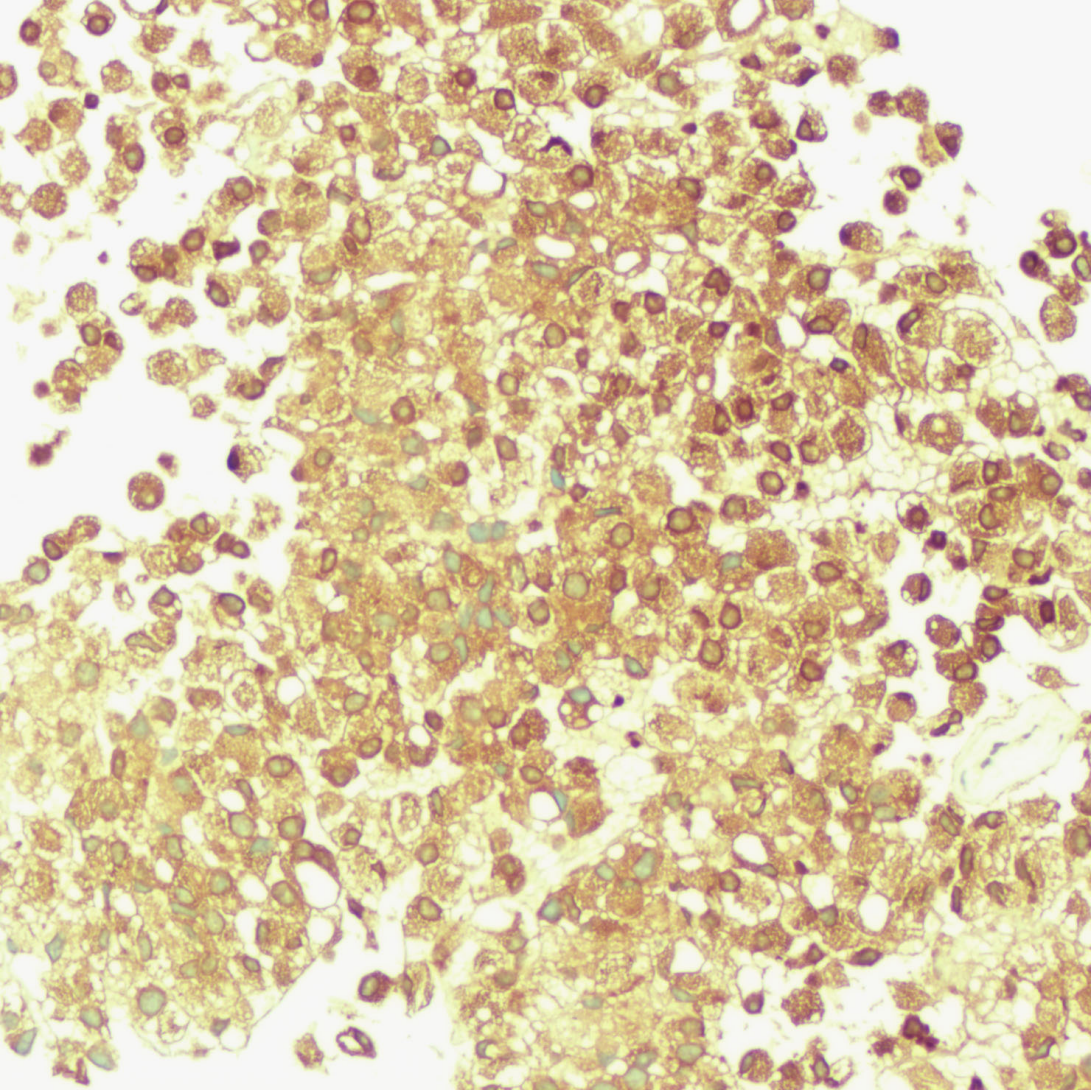
Immunohistochemistry showing PSA positivity in tumor cells.

The patient underwent a trans-pelvic mass puncture biopsy, yielding three strips of perforated tissue (7-14 mm in length and 1 mm in diameter). At low magnification, diffuse patches of signet-ring cells were observed within the punctured tissue (**Figure 5**). These cells varied in size, and a few hyperplastic vessels were present in the interstitium. Under high magnification (**Figure 6**), the nuclei were displaced to one side, presenting a signet-ring appearance. The tumor cell nuclei were large and deeply stained, with dense chromatin and irregular nuclear membranes. Some cells contained translucent large secretory mucus-like vesicles. Additionally, part of the cytoplasm was deeply stained and cloudy, with visible mucus-like vacuoles and clear cytoplasmic borders. Immunohistochemical tests suggested positivity for CK (**Figure 7**), P504S, PSA (**Figure 8**), CD138 (foci), and partial Ki-67 positivity (5%). The tests were negative for CDX-2, CK20, CK7, LCA, SATB2, CD56, CgA, INSM1, Syn, Vimentin, and PAS.

Based on the clinical history, imaging features, cytologic characteristics, histopathologic biopsy, and immunohistochemical results, the patient was pathologically diagnosed with primary prostate SRCC involving the pelvis.

The patient received chemoradiotherapy and has been followed up for three months. He is currently in good condition.

## Discussion

SRCCs are highly malignant, dedifferentiated adenocarcinomas containing numerous vesicles filled with mucin secreted from the cells. These tumors are poorly adherent and consist primarily of tumor cells. The cytoplasm contains a large amount of mucin, which displaces the nucleus to the edge of the cytoplasm, creating a “signet ring” appearance (4). SRCC is most commonly found in gastrointestinal tumors, accounting for approximately 35.45% of new gastric cancer cases (5). Besides gastric cancer, SRCC can also occur in other solid tumors, including colorectal, esophageal, and breast cancers, although these occurrences are relatively rare. SRCC involving the prostate is extremely rare (6). This report details an exceptionally rare case of primary prostate SRCC with pelvic involvement.

Prostate cancer is a relatively indolent malignant tumor of the reproductive system, prevalent in older men, characterized by a long disease course and slow onset. According to the fifth edition of the WHO Classification of Tumors of the Urological and Male Genital System (ICD:8490/3), the pathological types of prostate cancer include acinar adenocarcinoma, intraductal carcinoma, ductal adenocarcinoma, urothelial carcinoma, squamous cell carcinoma, basal cell carcinoma, and neuroendocrine tumors (7). The most common type of prostate malignancy is acinar adenocarcinoma, which accounts for approximately 90% of all prostate cancers. SRCC is extremely rare in prostate cancer, representing approximately 2.5% of all prostate cancers (8).

Patients with primary prostate SRCC typically present with bladder outlet obstruction symptoms such as dysuria, urinary frequency, nocturia, and urinary retention. Some patients may also experience bladder irritation symptoms and hematuria (9). Prostate SRCC is highly invasive, exhibiting diffuse infiltrative growth and often invading the neurovascular space and extraprostatic tissues. Most patients rapidly develop metastatic symptoms, including bone metastases and metastases to other sites, within a short period of time (10). Prostate SRCC is characterized by unique biological behavior, high malignancy, rapid disease progression, poor prognosis, and an extremely low five-year survival rate. Typically diagnosed at an advanced stage, this disease has an average survival rate of only 29 months (11). Due to its high invasiveness and propensity for distant metastasis, early diagnosis and treatment of primary prostate SRCC are crucial. In this case, the patient did not have obvious symptoms of urinary system diseases such as frequency, urgency, and hematuria. In contrast, the initial clinical symptoms were related to the digestive system, including abdominal pain, altered bowel habits, and bloody stool, which often prompted clinicians to initially consider and treat digestive system diseases, such as rectal cancer, during the first visit.

SRCC is a specialized type of adenocarcinoma characterized by the presence of abundant mucus within the cells. Microscopically, the mucus fills the cell, displacing the nucleus to one side and creating the characteristic signet-ring appearance.

The diagnosis of primary prostate SRCC relies primarily on pathohistologic examination. Prostate SRCC is usually mixed with components of classic prostate cancer. Under the optical microscope, it predominantly exhibits diffuse unicellular infiltration, characterized cytologically by large, rounded, or ovoid tumor cells with abundant hyaline cytoplasm and nuclei displaced to one side, often forming a signet-ring appearance. Electron microscopy reveals intracytoplasmic lumen and vacuole formation, typically without mucus and fat vacuoles. Immunohistochemical analysis reveals positive PSA and PAP staining in the cancer cytoplasm, predominantly negative CEA, and negative results for AB/PAS, LCA, and αSMA. Primary prostate SRCC is a rare histologic variant of prostate adenocarcinoma. Diagnosis necessitates that signet-ring cells constitute over 25% of the tumor (12), although another study proposes that an RSCC component exceeding 50% is required for diagnosis (13). Screening other tumor sites in the gastrointestinal tract is essential to exclude the possibility of gastrointestinal SRCC metastasis when diagnosing primary SRCC of the prostate.

In this case, the signet ring cell-like tumor cells, characterized by CK (+), P504S (+), and PSA (+), indicate a prostatic epithelial origin. The absence of CDX-2 (-), CK20 (-), CK7 (-), SATB2 (-), and PAS (-) rules out metastasis from signet ring cell carcinoma of the digestive system. Additionally, LCA (-) excludes lymphoid tumors, while CD56 (-), CgA (-), INSM1 (-), and Syn (-) eliminate the possibility of neuroendocrine tumors.

Currently, the treatment of prostate SRCC is similar to that of ordinary prostate cancer. Treatment options, including surgery, endocrine therapy, and chemoradiotherapy, should be carefully considered based on the patient’s condition (14). However, prostate SRCC is less sensitive to endocrine therapy (9). Radical surgical treatment remains a priority for eligible patients. As with conventional prostate cancer, radical surgery is the most effective treatment for early-stage prostate SRCC.

### Differential diagnosis

Cytology: SRCC should be differentiated from other diseases that feature cells with a signet-ring appearance, such as signet-ring mesothelial cells, signet-ring macrophages, and malignant signet-ring cells (including signet-ring adenocarcinoma cells and signet-ring carcinoma cells).

Morphological features of signet-ring mesothelial mesothelial cells (15): The cell body is markedly swollen and enlarged, round or round-like. The nucleus is regular in shape, squeezed to one side, often stratified, with coarse chromatin and infrequent nucleoli. The cytoplasm is dominated by degenerated large vacuoles, with uneven staining and very pale staining at the distal nuclear ends, but with clear cytoplasmic boundaries.

Morphological features of signet-ring macrophages (16): The cytosol size varies, with irregular and lateralized nuclei. The chromatin exhibits coarse reticulation, with nucleoli being infrequent. The cytoplasmic mass varies, with uniform staining. The cytoplasm contains vacuoles of varying sizes, and cytoplasmic borders may not be clear.

Morphological features of malignant signet-ring cells (17): (1) Signet-ring cytological appearance: Cells exhibit a characteristic signet-ring appearance; (2) Large cell body: Cells have a large body, either singly distributed or arranged in clusters, and may fuse with each other; (3) Cytosolic nuclei: Nuclei vary in size, may be more than one, are rounded or irregularly shaped, with fine dense chromatin and obvious nucleoli; (4) Cytoplasm: The cytoplasm appears deeply stained and cloudy, containing large mucus-secreting vacuoles that may coalesce.

Signet-ring adenocarcinoma cells (18): Large mucus-secreting vesicles within a single cell are crucial for the secretory function of adenocarcinoma cells. When these vesicles are enlarged, the nucleus is typically displaced to one side, imparting a signet-ring appearance to the cell.

Morphological features of signet-ring adenocarcinoma cells: The cell body is significantly enlarged, with the nucleus displaced to one side (sometimes more than one nucleus). The chromatin is dense and deeply stained, and the nucleolus is prominent. The cytoplasm is deeply stained and cloudy, with large mucus-secreting vacuoles that may fuse together, and the cytoplasmic boundary is clear.

Signet-ring carcinoma cells (19): These are a specialized type of malignant signet-ring adenocarcinoma cells, commonly known as signet-ring cells.

Morphological features of signet-ring carcinoma cells include: Rarely displaying the classic signet-ring cytological appearance; (2) Variable cell body size, often with a scattered distribution and infrequent fusion; (3) Enlarged and markedly displaced nuclei, sometimes multinucleated, with a rounded or orbicular shape; the side exhibiting the signet-ring morphology is typically flat. Chromatin is dense and unevenly distributed, with an abundant and prominent nucleolus; (4) Deeply stained cytoplasm containing numerous small mucus vacuoles, which may occasionally obscure the nucleus. In some cases, the cytoplasmic edge may exhibit tumorous protuberances.

Histology: Signet-ring changes of cells may also occur in benign reactive lesions. Signet-ring cells are predominantly found in the glandular cells of the stomach (SRCC originates in the stomach in 90% of patients) (20), and less commonly in the breast, gallbladder, bladder, and pancreas. The present case originated from the prostate, which is extremely rare and requires differential diagnosis using histologic morphology, immunohistochemistry, and potentially molecular testing.

The identification of signet-ring cells in conventional cell smears poses a significant challenge. They typically appear as single cells with vacuoles in the cytoplasm pushing the nucleus to one side. The relatively mild morphology of the nucleus, due to its low nucleoplasm ratio, poses a challenge for clinicopathologic diagnosis. Diagnosing SRCC in traditional puncture smears is challenging, and additional auxiliary examinations are necessary to confirm the diagnosis.

## Conclusion

This report presents a rare case of signet-ring cells observed in a conventional cytologic smear. Signet-ring cells were also identified in a histopathologic biopsy and diagnosed as prostate SRCC through immunohistochemistry and special staining. This report focuses on the morphological differences and differential diagnosis of signet-ring cells in cytological smears and histopathological biopsies. It also summarizes the clinical experience of diagnosing diseases associated with signet-ring cells using cytology. This is the first reported case of prostate SRCC initially presenting with gastrointestinal symptoms, which can enhance clinicians’ diagnostic approaches and help prevent misdiagnosis as digestive system conditions, such as rectal cancer, thereby holding significant value for clinical diagnosis.

## Data Availability

The original contributions presented in the study are included in the article/supplementary material. Further inquiries can be directed to the corresponding author.
